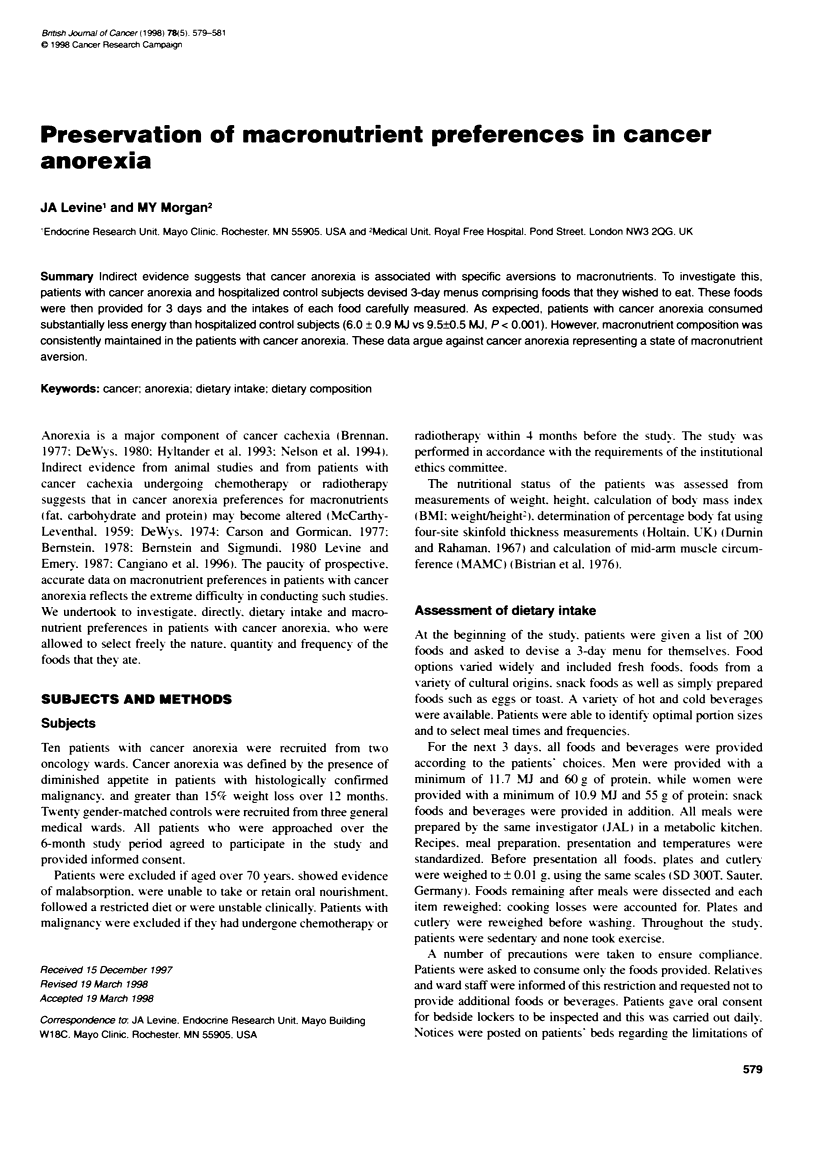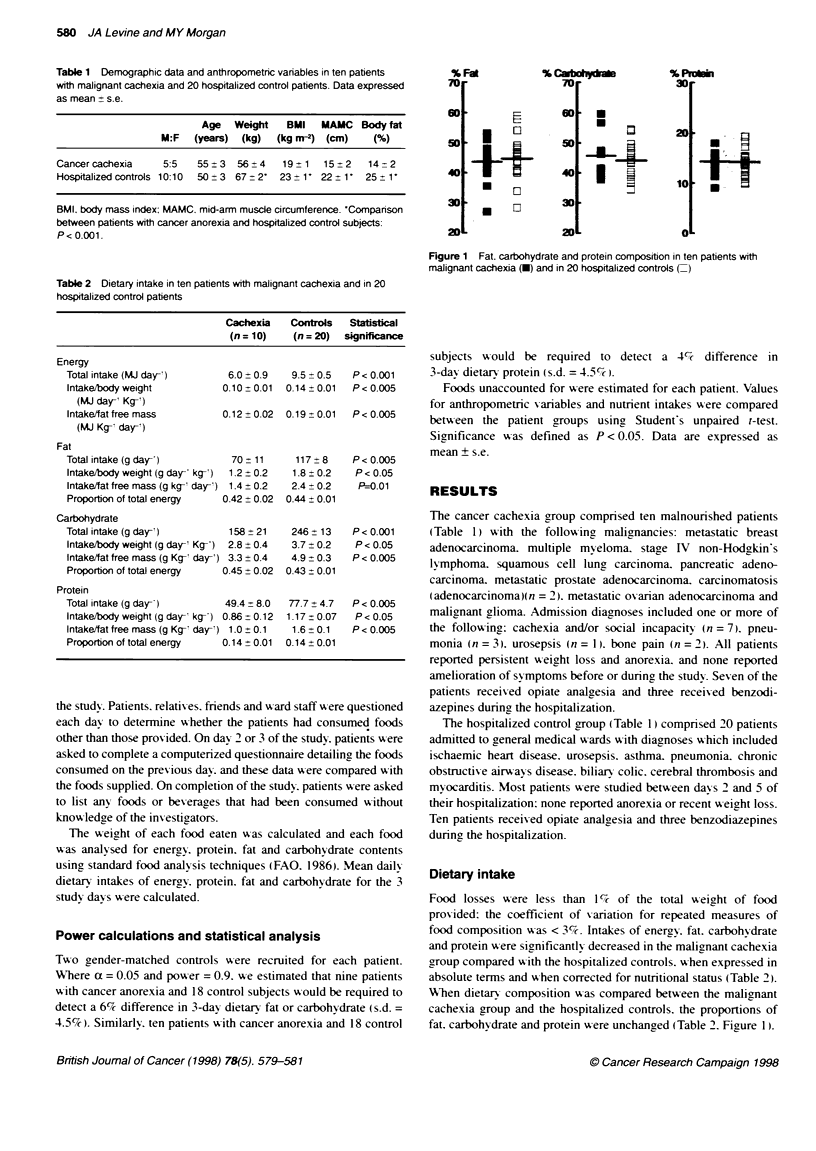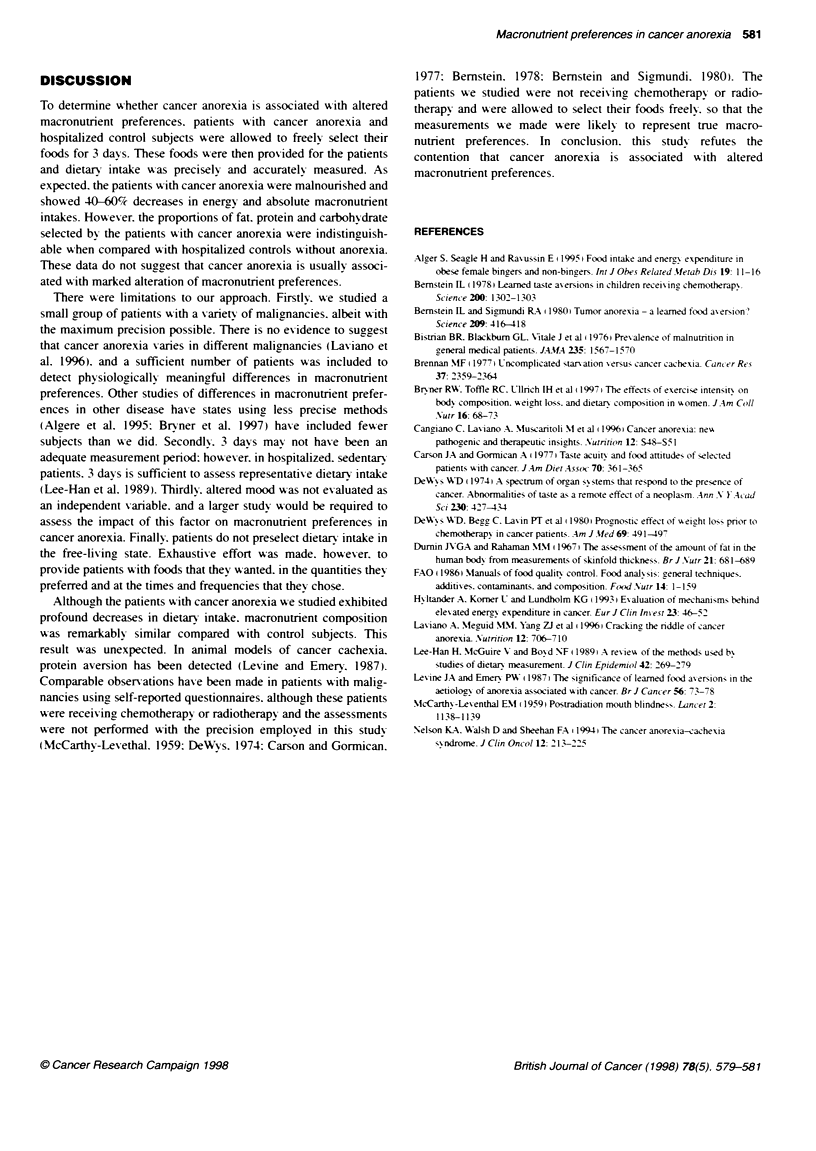# Preservation of macronutrient preferences in cancer anorexia.

**DOI:** 10.1038/bjc.1998.543

**Published:** 1998-09

**Authors:** J. A. Levine, M. Y. Morgan

**Affiliations:** Endocrine Research Unit, Mayo Clinic, Rochester, MN 55905, USA.

## Abstract

Indirect evidence suggests that cancer anorexia is associated with specific aversions to macronutrients. To investigate this, patients with cancer anorexia and hospitalized control subjects devised 3-day menus comprising foods that they wished to eat. These foods were then provided for 3 days and the intakes of each food carefully measured. As expected, patients with cancer anorexia consumed substantially less energy than hospitalized control subjects (6.0 +/- 0.9 MJ vs 9.5 +/- 0.5 MJ, P < 0.001). However, macronutrient composition was consistently maintained in the patients with cancer anorexia. These data argue against cancer anorexia representing a state of macronutrient aversion.


British Journal of Cancer (1998) 78(5). 579–581
© 1998 Cancer Research Campaign

### Preservation of macronutrient preferences in cancer anorexia

**JA Levine[1] and MY Morgan[2]**

[1]Endocrine Research Unit. Mayo Clinic. Rochester. MN 55905. USA and [2]Medical Unit. Royal Free Hospital. Pond Street. London NW3 2QG. UK

**Summary** Indirect evidence suggests that cancer anorexia is associated with specific aversions to macronutrients. To investigate this, patients with cancer anorexia and hospitalized control subjects devised 3-day menus comprising foods that they wished to eat. These foods were then provided for 3 days and the intakes of each food carefully measured. As expected, patients with cancer anorexia consumed substantially less energy than hospitalized control subjects (6.0 ± 0.9 MJ vs 9.5±0.5 MJ, $P < 0.001$). However, macronutrient composition was consistently maintained in the patients with cancer anorexia. These data argue against cancer anorexia representing a state of macronutrient aversion.

**Keywords:** cancer; anorexia; dietary intake; dietary composition

Anorexia is a major component of cancer cachexia (Brennan. 1977: DeWys. 1980: Hyltander et al. 1993: Nelson et al. 1994). Indirect evidence from animal studies and from patients with cancer cachexia undergoing chemotherapy or radiotherapy suggests that in cancer anorexia preferences for macronutrients (fat, carbohydrate and protein) may become altered (McCarthy-Leventhal. 1959: DeWys. 1974: Carson and Gormican. 1977: Bernstein. 1978: Bernstein and Sigmundi. 1980 Levine and Emery. 1987: Cangiano et al. 1996). The paucity of prospective, accurate data on macronutrient preferences in patients with cancer anorexia reflects the extreme difficulty in conducting such studies. We undertook to investigate, directly, dietary intake and macronutrient preferences in patients with cancer anorexia, who were allowed to select freely the nature, quantity and frequency of the foods that they ate.

### SUBJECTS AND METHODS

### Subjects

Ten patients with cancer anorexia were recruited from two oncology wards. Cancer anorexia was defined by the presence of diminished appetite in patients with histologically confirmed malignancy, and greater than 15% weight loss over 12 months. Twenty gender-matched controls were recruited from three general medical wards. All patients who were approached over the 6-month study period agreed to participate in the study and provided informed consent.

Patients were excluded if aged over 70 years, showed evidence of malabsorption, were unable to take or retain oral nourishment, followed a restricted diet or were unstable clinically. Patients with malignancy were excluded if they had undergone chemotherapy or radiotherapy within 4 months before the study. The study was performed in accordance with the requirements of the institutional ethics committee.

The nutritional status of the patients was assessed from measurements of weight, height, calculation of body mass index (BMI: weight/height$^2$), determination of percentage body fat using four-site skinfold thickness measurements (Holtain. UK) (Durnin and Rahaman. 1967) and calculation of mid-arm muscle circumference (MAMC) (Bistrian et al. 1976).

### Assessment of dietary intake

At the beginning of the study, patients were given a list of 200 foods and asked to devise a 3-day menu for themselves. Food options varied widely and included fresh foods, foods from a variety of cultural origins, snack foods as well as simply prepared foods such as eggs or toast. A variety of hot and cold beverages were available. Patients were able to identify optimal portion sizes and to select meal times and frequencies.

For the next 3 days, all foods and beverages were provided according to the patients' choices. Men were provided with a minimum of 11.7 MJ and 60 g of protein, while women were provided with a minimum of 10.9 MJ and 55 g of protein: snack foods and beverages were provided in addition. All meals were prepared by the same investigator (JAL) in a metabolic kitchen. Recipes, meal preparation, presentation and temperatures were standardized. Before presentation all foods, plates and cutlery were weighed to ± 0.01 g, using the same scales (SD 300T. Sauter. Germany). Foods remaining after meals were dissected and each item reweighed: cooking losses were accounted for. Plates and cutlery were reweighed before washing. Throughout the study, patients were sedentary and none took exercise.

A number of precautions were taken to ensure compliance. Patients were asked to consume only the foods provided. Relatives and ward staff were informed of this restriction and requested not to provide additional foods or beverages. Patients gave oral consent for bedside lockers to be inspected and this was carried out daily. Notices were posted on patients' beds regarding the limitations of

*Received 15 December 1997*
*Revised 19 March 1998*
*Accepted 19 March 1998*

*Correspondence to:* JA Levine. Endocrine Research Unit. Mayo Building W18C. Mayo Clinic. Rochester. MN 55905. USA

**Table 1** Demographic data and anthropometric variables in ten patients with malignant cachexia and 20 hospitalized control patients. Data expressed as mean ± s.e.

| | M:F | Age (years) | Weight (kg) | BMI (kg m⁻²) | MAMC (cm) | Body fat (%) |
|---|---|---|---|---|---|---|
| Cancer cachexia | 5:5 | 55 ± 3 | 56 ± 4 | 19 ± 1 | 15 ± 2 | 14 ± 2 |
| Hospitalized controls | 10:10 | 50 ± 3 | 67 ± 2* | 23 ± 1* | 22 ± 1* | 25 ± 1* |

BMI, body mass index; MAMC, mid-arm muscle circumference. *Comparison between patients with cancer anorexia and hospitalized control subjects: $P < 0.001$.

**Figure 1** Fat, carbohydrate and protein composition in ten patients with malignant cachexia (■) and in 20 hospitalized controls (□)

**Table 2** Dietary intake in ten patients with malignant cachexia and in 20 hospitalized control patients

| | Cachexia ($n$ = 10) | Controls ($n$ = 20) | Statistical significance |
|---|---|---|---|
| Energy | | | |
| Total intake (MJ day⁻¹) | 6.0 ± 0.9 | 9.5 ± 0.5 | $P < 0.001$ |
| Intake/body weight (MJ day⁻¹ Kg⁻¹) | 0.10 ± 0.01 | 0.14 ± 0.01 | $P < 0.005$ |
| Intake/fat free mass (MJ Kg⁻¹ day⁻¹) | 0.12 ± 0.02 | 0.19 ± 0.01 | $P < 0.005$ |
| Fat | | | |
| Total intake (g day⁻¹) | 70 ± 11 | 117 ± 8 | $P < 0.005$ |
| Intake/body weight (g day⁻¹ kg⁻¹) | 1.2 ± 0.2 | 1.8 ± 0.2 | $P < 0.05$ |
| Intake/fat free mass (g kg⁻¹ day⁻¹) | 1.4 ± 0.2 | 2.4 ± 0.2 | $P$=0.01 |
| Proportion of total energy | 0.42 ± 0.02 | 0.44 ± 0.01 | |
| Carbohydrate | | | |
| Total intake (g day⁻¹) | 158 ± 21 | 246 ± 13 | $P < 0.001$ |
| Intake/body weight (g day⁻¹ Kg⁻¹) | 2.8 ± 0.4 | 3.7 ± 0.2 | $P < 0.05$ |
| Intake/fat free mass (g Kg⁻¹ day⁻¹) | 3.3 ± 0.4 | 4.9 ± 0.3 | $P < 0.005$ |
| Proportion of total energy | 0.45 ± 0.02 | 0.43 ± 0.01 | |
| Protein | | | |
| Total intake (g day⁻¹) | 49.4 ± 8.0 | 77.7 ± 4.7 | $P < 0.005$ |
| Intake/body weight (g day⁻¹ kg⁻¹) | 0.86 ± 0.12 | 1.17 ± 0.07 | $P < 0.05$ |
| Intake/fat free mass (g Kg⁻¹ day⁻¹) | 1.0 ± 0.1 | 1.6 ± 0.1 | $P < 0.005$ |
| Proportion of total energy | 0.14 ± 0.01 | 0.14 ± 0.01 | |

the study. Patients, relatives, friends and ward staff were questioned each day to determine whether the patients had consumed foods other than those provided. On day 2 or 3 of the study, patients were asked to complete a computerized questionnaire detailing the foods consumed on the previous day, and these data were compared with the foods supplied. On completion of the study, patients were asked to list any foods or beverages that had been consumed without knowledge of the investigators.

The weight of each food eaten was calculated and each food was analysed for energy, protein, fat and carbohydrate contents using standard food analysis techniques (FAO, 1986). Mean daily dietary intakes of energy, protein, fat and carbohydrate for the 3 study days were calculated.

### Power calculations and statistical analysis

Two gender-matched controls were recruited for each patient. Where $\alpha = 0.05$ and power = 0.9, we estimated that nine patients with cancer anorexia and 18 control subjects would be required to detect a 6% difference in 3-day dietary fat or carbohydrate (s.d. = 4.5%). Similarly, ten patients with cancer anorexia and 18 control subjects would be required to detect a 4% difference in 3-day dietary protein (s.d. = 4.5%).

Foods unaccounted for were estimated for each patient. Values for anthropometric variables and nutrient intakes were compared between the patient groups using Student's unpaired $t$-test. Significance was defined as $P < 0.05$. Data are expressed as mean ± s.e.

### RESULTS

The cancer cachexia group comprised ten malnourished patients (Table 1) with the following malignancies: metastatic breast adenocarcinoma, multiple myeloma, stage IV non-Hodgkin's lymphoma, squamous cell lung carcinoma, pancreatic adenocarcinoma, metastatic prostate adenocarcinoma, carcinomatosis (adenocarcinoma)($n$ = 2), metastatic ovarian adenocarcinoma and malignant glioma. Admission diagnoses included one or more of the following: cachexia and/or social incapacity ($n$ = 7), pneumonia ($n$ = 3), urosepsis ($n$ = 1), bone pain ($n$ = 2). All patients reported persistent weight loss and anorexia, and none reported amelioration of symptoms before or during the study. Seven of the patients received opiate analgesia and three received benzodiazepines during the hospitalization.

The hospitalized control group (Table 1) comprised 20 patients admitted to general medical wards with diagnoses which included ischaemic heart disease, urosepsis, asthma, pneumonia, chronic obstructive airways disease, biliary colic, cerebral thrombosis and myocarditis. Most patients were studied between days 2 and 5 of their hospitalization; none reported anorexia or recent weight loss. Ten patients received opiate analgesia and three benzodiazepines during the hospitalization.

### Dietary intake

Food losses were less than 1% of the total weight of food provided; the coefficient of variation for repeated measures of food composition was < 3%. Intakes of energy, fat, carbohydrate and protein were significantly decreased in the malignant cachexia group compared with the hospitalized controls, when expressed in absolute terms and when corrected for nutritional status (Table 2). When dietary composition was compared between the malignant cachexia group and the hospitalized controls, the proportions of fat, carbohydrate and protein were unchanged (Table 2, Figure 1).

© Cancer Research Campaign 1998

### DISCUSSION

To determine whether cancer anorexia is associated with altered macronutrient preferences, patients with cancer anorexia and hospitalized control subjects were allowed to freely select their foods for 3 days. These foods were then provided for the patients and dietary intake was precisely and accurately measured. As expected, the patients with cancer anorexia were malnourished and showed 40–60% decreases in energy and absolute macronutrient intakes. However, the proportions of fat, protein and carbohydrate selected by the patients with cancer anorexia were indistinguishable when compared with hospitalized controls without anorexia. These data do not suggest that cancer anorexia is usually associated with marked alteration of macronutrient preferences.

There were limitations to our approach. Firstly, we studied a small group of patients with a variety of malignancies, albeit with the maximum precision possible. There is no evidence to suggest that cancer anorexia varies in different malignancies (Laviano et al. 1996), and a sufficient number of patients was included to detect physiologically meaningful differences in macronutrient preferences. Other studies of differences in macronutrient preferences in other disease have states using less precise methods (Algere et al. 1995; Bryner et al. 1997) have included fewer subjects than we did. Secondly, 3 days may not have been an adequate measurement period; however, in hospitalized, sedentary patients, 3 days is sufficient to assess representative dietary intake (Lee-Han et al. 1989). Thirdly, altered mood was not evaluated as an independent variable, and a larger study would be required to assess the impact of this factor on macronutrient preferences in cancer anorexia. Finally, patients do not preselect dietary intake in the free-living state. Exhaustive effort was made, however, to provide patients with foods that they wanted, in the quantities they preferred and at the times and frequencies that they chose.

Although the patients with cancer anorexia we studied exhibited profound decreases in dietary intake, macronutrient composition was remarkably similar compared with control subjects. This result was unexpected. In animal models of cancer cachexia, protein aversion has been detected (Levine and Emery, 1987). Comparable observations have been made in patients with malignancies using self-reported questionnaires, although these patients were receiving chemotherapy or radiotherapy and the assessments were not performed with the precision employed in this study (McCarthy-Levethal, 1959; DeWys, 1974; Carson and Gormican, 1977; Bernstein, 1978; Bernstein and Sigmundi, 1980). The patients we studied were not receiving chemotherapy or radiotherapy and were allowed to select their foods freely, so that the measurements we made were likely to represent true macronutrient preferences. In conclusion, this study refutes the contention that cancer anorexia is associated with altered macronutrient preferences.

### REFERENCES

Alger S, Seagle H and Ravussin E (1995) Food intake and energy expenditure in obese female bingers and non-bingers. *Int J Obes Related Metab Dis* **19**: 11–16

Bernstein IL (1978) Learned taste aversions in children receiving chemotherapy. *Science* **200**: 1302–1303

Bernstein IL and Sigmundi RA (1980) Tumor anorexia – a learned food aversion? *Science* **209**: 416–418

Bistrian BR, Blackburn GL, Vitale J et al (1976) Prevalence of malnutrition in general medical patients. *JAMA* **235**: 1567–1570

Brennan MF (1977) Uncomplicated starvation versus cancer cachexia. *Cancer Res* **37**: 2359–2364

Bryner RW, Toffle RC, Ullrich IH et al (1997) The effects of exercise intensity on body composition, weight loss, and dietary composition in women. *J Am Coll Nutr* **16**: 68–73

Cangiano C, Laviano A, Muscaritoli M et al (1996) Cancer anorexia: new pathogenic and therapeutic insights. *Nutrition* **12**: S48–S51

Carson JA and Gormican A (1977) Taste acuity and food attitudes of selected patients with cancer. *J Am Diet Assoc* **70**: 361–365

DeWys WD (1974) A spectrum of organ systems that respond to the presence of cancer. Abnormalities of taste as a remote effect of a neoplasm. *Ann NY Acad Sci* **230**: 427–434

DeWys WD, Begg C, Lavin PT et al (1980) Prognostic effect of weight loss prior to chemotherapy in cancer patients. *Am J Med* **69**: 491–497

Durnin JVGA and Rahaman MM (1967) The assessment of the amount of fat in the human body from measurements of skinfold thickness. *Br J Nutr* **21**: 681–689

FAO (1986) Manuals of food quality control. Food analysis: general techniques, additives, contaminants, and composition. *Food Nutr* **14**: 1–159

Hyltander A, Korner U and Lundholm KG (1993) Evaluation of mechanisms behind elevated energy expenditure in cancer. *Eur J Clin Invest* **23**: 46–52

Laviano A, Meguid MM, Yang ZJ et al (1996) Cracking the riddle of cancer anorexia. *Nutrition* **12**: 706–710

Lee-Han H, McGuire V and Boyd NF (1989) A review of the methods used by studies of dietary measurement. *J Clin Epidemiol* **42**: 269–279

Levine JA and Emery PW (1987) The significance of learned food aversions in the aetiology of anorexia associated with cancer. *Br J Cancer* **56**: 73–78

McCarthy-Leventhal EM (1959) Postradiation mouth blindness. *Lancet* **2**: 1138–1139

Nelson KA, Walsh D and Sheehan FA (1994) The cancer anorexia–cachexia syndrome. *J Clin Oncol* **12**: 213–225

© Cancer Research Campaign 1998